# Complete genome sequence of a winter season *Vibrio* facilitates discovery of a novel subclade of cold-adapted species in the *albus* clade

**DOI:** 10.1099/mgen.0.001178

**Published:** 2024-01-17

**Authors:** Paul D. Kastner, Stephen E. Noell, David A. Essig

**Affiliations:** ^1^​ School of Medicine, Vanderbilt University, Nashville, TN, USA; ^2^​ Te Aka Mātuatua – School of Science, Thermophile Research Unit, Te Whare Wānanga o Waikato – University of Waikato, Hamilton, New Zealand; ^3^​ Department of Biology, Geneva College, Beaver Falls, PA, USA

## Abstract

In temperate marine climate zones, seasonal changes in water temperature contribute to distinct populations of warm- and cold-water vibrios. We report here the complete genome sequence (BUSCO score=94.8) of the novel strain *Vibrio* sp. VB16 isolated in late winter from the intertidal zone near Virginia Beach, Virginia, USA with the ability to form colonies at 4 °C. The 5.2 Mbp genome is composed of a large (3.6 Mbp) and small (1.6 Mbp) chromosome. Based on paired average nucleotide identity (ANI), average amino acid identity (AAI) and digital DNA–DNA hybridization (dDDH), *V.* sp. VB16 is the same species as *V*. sp. UBA2437 from a North Sea tidal flat and is closely related to *V*. sp. DW001 from Antarctic sea ice. Our phylogenomic and bioinformatic analyses placed VB16, UBA2437 and DW001 into a cold-tolerant subclade within the *albus* clade, along with two non-cold-tolerant subclades. Orthovenn analysis indicated that VB16 and its other *albus* clade members shared 1544 gene orthologue clusters, including clusters for biosynthesis of polar flagella and tight adhesion pili that predict multiple lifestyles, either free-living or as an opportunistic pathogen within a marine eukaryotic host. The cold-tolerant subclade shared 552 orthologue proteins, including genes known to promote survival in cold or freezing temperatures, such as the eicosapentaenoic acid biosynthetic gene cluster, *syp* exopolysaccharide gene cluster and novel giant proteins with ice-binding domains. This subclade represents a group of psychrotolerant or ‘moderate psychrophile’ winter season *Vibrio* species. The discovery of this subclade opens the door for experimental work on the physiological features, virulence potential and ecological importance of this subclade.

## Abbreviations

AAI, average amino acid identity; ANI, average nucleotide identity; BLASTN, Basic Local Alignment Search Tool - Nucleotide; BLASTP, Basic Local Alignment Search Tool - Protein; BMC, bacterial microcompartment; dDDH, digital DNA-DNA hybridization; *eut*, ethanolamine utilization; GTDB, genome taxonomy database; NCBI, National Center for Biotechnology Information; OGT, optimal growth temperature; PATRIC, pathosystems resource integration center; RASTtk, Rapid Annotatioin using Subsytem Technology tool kit; *syp*, symbiosis polysaccharide.

## Data Summary

The genome assembly data are available in GenBank under accession numbers CPO87590.1 (Chr 1) and CP087591.1 (Chr 2). The Sequence Read Archive (SRA) accession numbers for the Illumina libraries and the MinION library are SRX9622697, SRX13131463 and SRX9622696, respectively.

### Impact Statement

We present the genome of novel cold-tolerant strain *Vibrio* sp. VB16, isolated from coastal Atlantic Ocean intertidal sediment during the winter season. Phylogenomic analyses predicted that this strain, along with *Vibrio* strain UBA2437, constitute a new species, which was placed with a related *Vibrio* strain DW001, in a new cold-tolerant subclade in the *albus* clade. This subclade consists of cold-adapted vibrios, as evidenced by multiple genomic hallmarks of psychrotolerant or ‘moderate psychrophile’ species, which correlated with measured and predicted optimal growth temperatures for VB16 (~20 °C), and were lower than those for the clade founding member *Vibrio albus* and other clade members. The vibrios within this subclade possess genomes adapted to either free-living or as an opportunistic pathogen within a marine eukaryotic host. The discovery of this subclade opens the door for experimental work to establish the physiological features, host virulence and ecological importance.

## Introduction

In temperate coastal marine environments, vibrios form biofilms on particle surfaces and play an important role in carbon and nitrogen cycling [1, 2]. Many vibrios are opportunistic pathogens of fish, shellfish and mammals, including humans [3]. There is seasonal variance in the abundance of *Vibrio* populations, with past evidence showing distinct communities in summer and winter months [4]. Many psychrophilic species thought to be vibrios have been reclassified into the genus *Aliivibrio* based on phylogenetic evidence (e.g. *Aliivibrio wodanis*) [5]. This raises the possibility that during the winter months cold-tolerant *Vibrio* species may become more abundant and take on greater ecological relevance. The degree to which such vibrios contribute to the rate of nutrient cycling in winter months has not been explored, in part due to the lack of a clearly defined winter season *Vibrio* species.

A survey of the temperature growth range of coastal vibrios demonstrated that most were mesophilic, although a few species (*Vibrio orientalis*, and two strains from *Vibrio splendidus*) were intermediate between psychrophilic and mesophilic, with maximal growth temperatures around 30 °C but the ability to grow at 4 °C [6]. True psychrophilic species of *Vibrio* that exhibit maximum growth at lower temperatures have been isolated from deep sea marine sediment but have not been characterized by genome analysis or taxonomically placed [7]. Low-temperature growth in these species of *Vibrio* correlated to the synthesis of membrane polyunsaturated fatty acids, either docosahexaenoic acid or eicosapentaenoic acid. Towards the goal of characterizing winter season *Vibrio* species, we screened bacteria isolated from late winter coastal sediment able to grow in plate culture at 4 °C. Based on 16S rRNA sequencing of the isolates, we identified a novel *Vibrio* strain VB16.

Here, we report the complete genome sequence of *V*. sp. VB16 (VB16), a putative winter season *Vibrio*. Phylogenomic analyses place it, along with two other sequenced *Vibrio* strains, into a new cold-tolerant subclade in the *albus* clade. Our analyses identified shared orthologue genes responsible for seasonal cold tolerance as well as a biofilm lifestyle either attached to particles or ice or within the intestinal tract of a marine animal host.

## Methods

### Isolation, growth conditions and genomic DNA isolation

A panel of cold-tolerant bacteria was isolated from the intertidal zone near Virginia Beach and PCR amplification/16S rRNA sequencing was performed by GENEWIZ (South Plainfield, NJ, USA) using previously described methods [8]. Based on pairwise blastn alignments of the 16S rRNA gene in EZBiocloud, the VB16 isolate was most closely related to *Vibrio ganglei* (95.2 % sequence identity).

### Optimal growth temperature

Optimal growth temperature (OGT) was determined for VB16 by comparing the time to reach stationary phase (or maximum cell density) when marine broth cultures were incubated at 4, 10, 20, 25 and 30 °C. Briefly, 0.5 ml of an overnight mid-log culture (0.6 A_600_) was inoculated into 50 ml of marine broth and shaken at 150 r.p.m. for varying time intervals in order achieve early stationary phase at all temperatures. OGT was also predicted from proteome sequences using TOME [9].

### Genomic sequencing, assembly and annotation

Frozen bacterial cell pellets of VB16 were prepared using the technique described elsewhere [8] and were sent to the MiGS Center (Pittsburgh, PA, USA) for sequencing. Genomic DNA was purified using the DNAeasy Blood and Tissue kit from Qiagen (Hilden, Germany) according to the user instructions.

An Illumina Nextera XT library was generated and sequenced using the NextSeq 2000 platform (2×151 bp; Illumina, San Diego, CA, USA). Sequence data analysis used default parameters unless otherwise specified. Total paired-end reads equaled 2 814 112 and yielded 759 097 985 bp. Quality control and adapter trimming were performed with bclfastq v.20.0.445 using default parameters. A Nanopore library was preprared using ligation kit SQK-LSK109 (Oxford Nanopore Technologies, Oxford, UK), bar-coded and sequenced in a MinION flow cell (v9.4, FLO-MIN106) using MinKNOW (v19.10.1) with Guppy (v4.2.2) as base caller (default settings+effbaf8). There were 313 545 reads totaling 2 388 935 290 bp. Quality control and adapter trimming were performed with Porechop v.0.2.3_sequan 2.1.1 using default parameters [10]. Hybrid assembly with Illumina and Nanopore reads was performed with Unicycler v0.4.8 [11], resulting in 12 contigs and a total length of 5 202 587 bp.

To improve the completeness of the genome sequence, a second Illumina library was generated using freshly isolated gDNA and sequenced in an identical manner to the first Illumina library. This library was combined with the first and yielded a total of 8 544 376 read pairs and 2 480 897 031 bp. Hybrid assembly using both sets of Illumina reads and Nanopore reads was performed with Unicycler v0.4.8 [11] and resulted in two contigs of 3  636 985 bp and 1 573 118 bp with 604× coverage. Each contig was able to circularize, consistent with the two (large and small) chromosome genome of most vibrios [12]. The total genome length was 5 209 308 bp.

The assembled genome quality was assessed using QUAST v.5.0.2 [13] and BUSCO v.5.3.2 [14] implemented on the Galaxy web platform [15]. Annotation of the assembled genome was performed in the Rapid Annotation using Subsystem Technology (RASTtk) platform [16].

### Phylogeny

To predict the closest genome neighbours to VB16 several approaches were employed. Two database programs were used that included the Similar Genome Finder [17] hosted in PATRIC [18], which utilizes a Mash/MinHash algorithm and the AAI profiler [19], which searched for proteomes in the UniProt database with the highest average amino acid identity (AAI) score and fractional representation compared to VB16. We also identified related *Vibrio* species based on blast hits in the National Center for Biotechnology Information (NCBI) database using amino acid sequences of VB16 for eight conserved *Vibrio* genes [20], and screening BioSample metadata for genome sequenced vibrios isolated from polar regions.

To screen for putative clade boundaries, an AAI heat map was generated using input genomes representing closely related clades from the *Vibrio* 3.0 classification [20] with EzAAI v1.2.2 [21]. We also included species genomes that represented orphan *Vibro* clades. The genomes used in this analysis are listed in Table S1, available in the online version of this article. The resulting heat map was clustered and visualized using the pheat map R package with the ‘maximum’ clustering method [22].

A *Vibrio* phylogenomic tree was produced using GToTree v1.6.34 [23] with the prepackaged single-copy gene set for Gammaproteobacteria. Genes were predicted from input genomes (the same set used for the AAI heat map, which included VB16 and those listed in Table S1 plus all *Vibrio* and *Aliivibrio* genomes in the Genome Taxonomy Database, GTDB) (provided as FASTA files) using Prodigal v2.6.3 [24]. Target genes (170) were identified with HMMER3 v3.2.2 [25], then aligned with Muscle v5.1 [26] and trimmed with Trimal v1.4.rev15 [27]. Sequences were finally concatenated, with a length of 38 650 bp from each genome of the concatenated, aligned sequences. Phylogenetic estimation was performed with default options in FastTree2 v2.1.11 [28], which used JTT, an approximately maximum-likelihood model (optimal for very large sequences [28]) and local support values to estimate the reliability of each split in the tree. The JTT model returned nearly identical Bayesian information criterion (BIC) scores to the recommended model (LG+I+G4) by IQ-Model Tree Finder [29], 1248801.0024 vs 1258736.4244, respectively. IQ-Model Finder was run with default parameters. TaxonKit [30] was used to connect full lineages to taxonomic IDs. The resulting tree was visualized using the interactive tree of life (iTOL, v6) (https://itol.embl.de/). *Photobacterium swingsii* was used as the outgroup.

A second *Vibrio* phylogenomic tree was generated using the web based autoMLST pipeline [31]. In this utility the set of database genomes was selected (Denovo mode) based on the input genomes via ANI estimation. Input genomes included *Vibro* strains VB16, UBA2437, DW001, 99-8-1, JC009, SCSIO 43137, F6 and F10, and species *Vibro maerli* and *Vibro albus*. A set of 78 single-copy genes appropriate for multilocus sequence comparison was selected, trimmed, concatenated and used for *de novo* tree building. Model finder was used to find the optimal model for building and bootstrap analysis (1000 replicates) performed by IQ Tree. *Saccharophagus degradans* was selected as the outgroup.

### Comparative genomics

Pairwise average nucleotide identity (ANI) and AAI scores were calculated using the online utilities of the Environmental Microbial Genomics Laboratory [32]. Digital DNA–DNA hybridization (dDDH) scores (Eq. 2) were obtained using the TYGS platform for genome-based taxonomy [33]. To compare the gene orthology of the species and strains most closely related to VB16, we used COGclassifier v1.0.5 [34]. The Orthovenn tool [35] set to a cutoff value (E-value) of 1e-10 was used to characterize the shared orthologues between VB16 and its related strains and species.

## Results and discussion

### Optimal growth temperature and cold growth phenotype

**Fig. 1.**
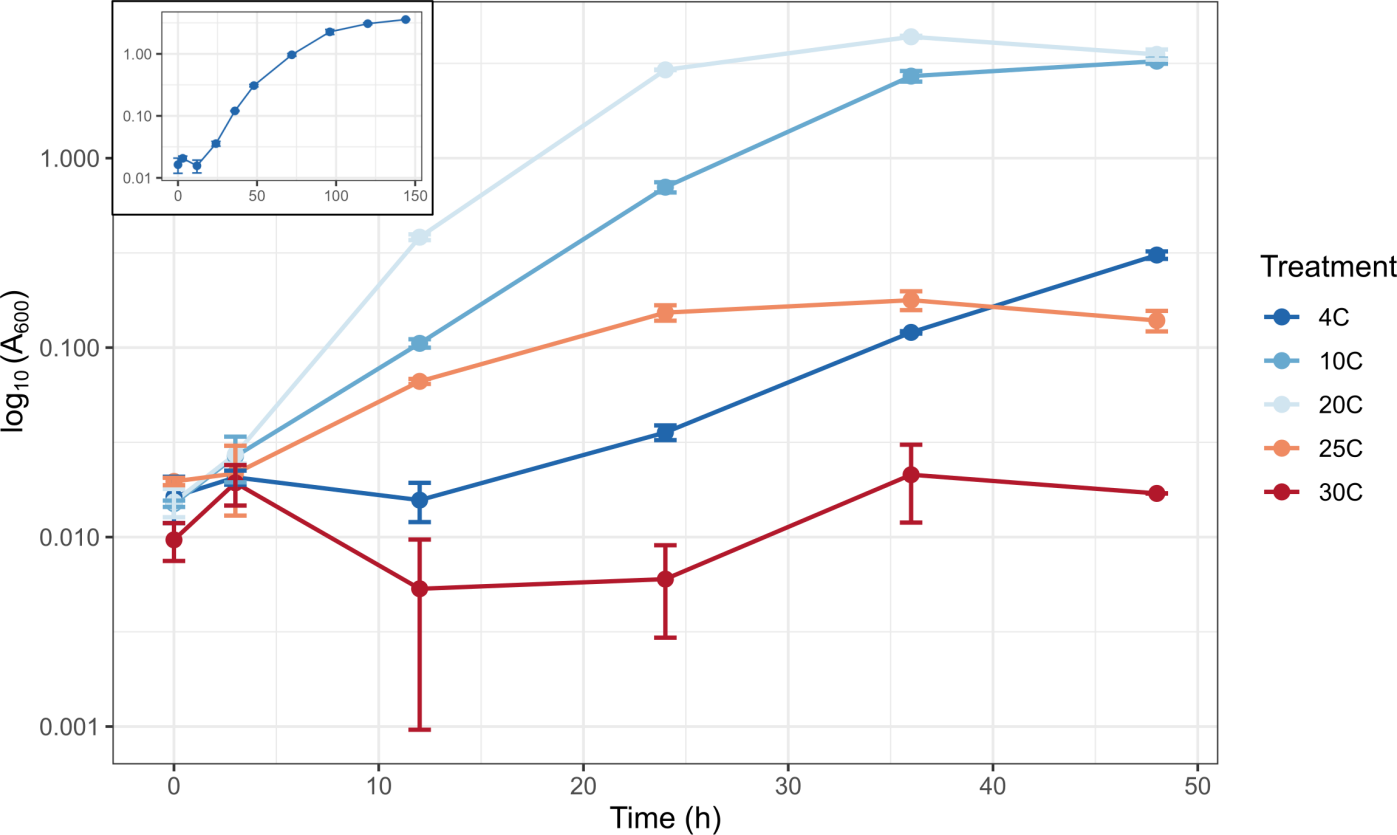
Cell density values [log_10_ (Abs600)] at selected time points for marine broth cultures of VB16 grown at 4, 10, 20, 25 and 30 °C for 48 h or 144 h at 4 °C (for all time points, see inset). Average values and SEM (error bars) are from *n*=3 replicate cultures. Where error bars are not visible, the SEM is very small and obscured by the data point.

We compared growth of VB16 in marine broth at 4, 10, 20, 25 and 30 °C. At 20 °C, maximal cell density was achieved by 36 h (Fig. 1). At 25 °C, growth reached peak density at 36 h but at a value ~20-fold less than density at 20 °C. Thereafter, growth declined further by 48 h. At 30 °C, there was no measurable growth. Growth at 10 °C was robust and by 36–48 h reached a similar density to the 20 °C optimal condition. The 4 °C condition (Fig. 1, inset) was characterized by a prolonged lag phase (~24 h). Thereafter, the growth rate was sustained, and eventually reached a stationary phase with similar cell density to that exhibited in the 10 and 20 °C conditions. While 20 °C was optimal for growth, VB16 has the ability to adapt during lag phase and demonstrate sustained growth at temperatures <20 °C.

### Genome assembly and features

QUAST analysis of the VB16 genome sequence (Table S2) indicated two circularized contigs resulting in a total length of 5 210 103 bp. The assembly had an N50 equal in size to the largest of the two contigs (3 636 985 bp), similar to the size of the large circular chromosome (chromosome 1; Table 1) typically found in vibrios [12]. The second contig equal to the N75 was 1 573 118 bp and corresponded to the small chromosome (chromosome 2; Table 1). The sequencing quality was high (Ns per 100 Kbp=0) and the BUSCO analysis (Table S1) gave a genome completeness score of 98.5 %.

**Table 1.** Summary of major features for each chromosome of VB16

Features	Chromosome 1	Chromosome 2
Length (bp)	3 636 985	1 573 118
GC content (%)	41.9	41.8
Protein coding genes/hypothetical	3340/722	1409/439
rRNA genes	28	0
tRNA genes	95	1
Genomic islands	5	3
Prophage insertion	1	0

### Genome annotation of VB16

The large and small chromosomes of VB16 had similar %GC content of 41.9 and 41.7, respectively (Table 1). These values are less than reported for *Vibrio cholerae* genomes (~46 %) but greater than *Vibrio fischeri* (~37 %) [36]. Based on RASTtk annotation, chromosome 1 contains 3341 protein coding genes (772 hypotheticals), while chromosome 2 contains 1409 genes (439 hypotheticals). All the rRNA and tRNA genes were found on chromosome 1, except for one tRNA in chromosome 2 (Table 1). It is noteworthy that chromosome 2s from most of the related *Vibrio* genomes (identified below) also possess only one tRNA gene. A total of eight genomic islands were detected by Island Viewer, five on chromosome 1 and three on chromosome 2. None bore any resemblance to pathogenicity islands typically associated with virulence in *V. cholerae* strains, such as VP-1, VP-2, VSP I, or VSP II [37]. A partial *Aeromonas phi018* prophage insertion on chromosome 1 was identified by PHASTER. We found no evidence of an integron integrase gene (IntI) or super integron on chromosome 2 as in *V. cholerae* strains [38].

### Genomes related to VB16

The closest relative identified was *Vibrio* strain UBA2437 (MASH distance of 0.03), sequenced from a North Sea tidal flat sediment sample [39, 40]. The only other *Vibrio* genome with a paired AAI value >76 % with VB16 was DW001, isolated from Antarctic sea ice. Single-value genome identity metrics including ANI, AAI and digital DNA–DNA hybridization (dDDH, Eq. 2) indicated that the genomes from VB16 and UBA2437 were strains of the same species (ANI and AAI >95 % and dDDH >75 %) (Table 2). DW001 was either another strain of the same species as VB16 and UBA2437 (based on AAI >95 %) or a strain from a highly related species (AAI=93.8 % and eDDH=56.9 % to VB16). Genome features were highly similar across these strains, with genome size the most variable (5.1 to 5.5 Mb) (Table 2).

**Table 2.** *Vibrio* genomes most closely related to VB16. All paired ANI, AAI and dDDH values are for the given strain compared to VB16

*Vibrio* strain	VB16	UBA2437	DW001
Isolation location	Intertidal sediment, Atlantic Ocean, Virginia, USA	tidal flat sediment, North Sea, Germany	sea ice, Southern Ocean, Antarctica
Climate zone	Temperate	Temperate	Polar
Genome size (Mbp)	5.2 Chr 1=3.6 Chr 2=1.6	4.6 (MAG=90 % complete); 5.1 after adjustment	5.5 Chr 1=3.8 Chr 2=1.7
GC (%)	41.9	41.9	41.8
Protein coding genes/hypothetical (#)	4749/1161	4745/1231	5098/1155
Paired ANI (%)		97.1	93.8
Paired AAI (%)		97.3	96.1
Paired dDDH (%)		76.4	56.9

Chr 1, chromosome 1; Chr 2, chromosome 2.

#### *Vibrio* strains VB16, UBA2437 and DW001 define a cold-tolerant subclade within an expanded *albus* clade

**Fig. 2.**
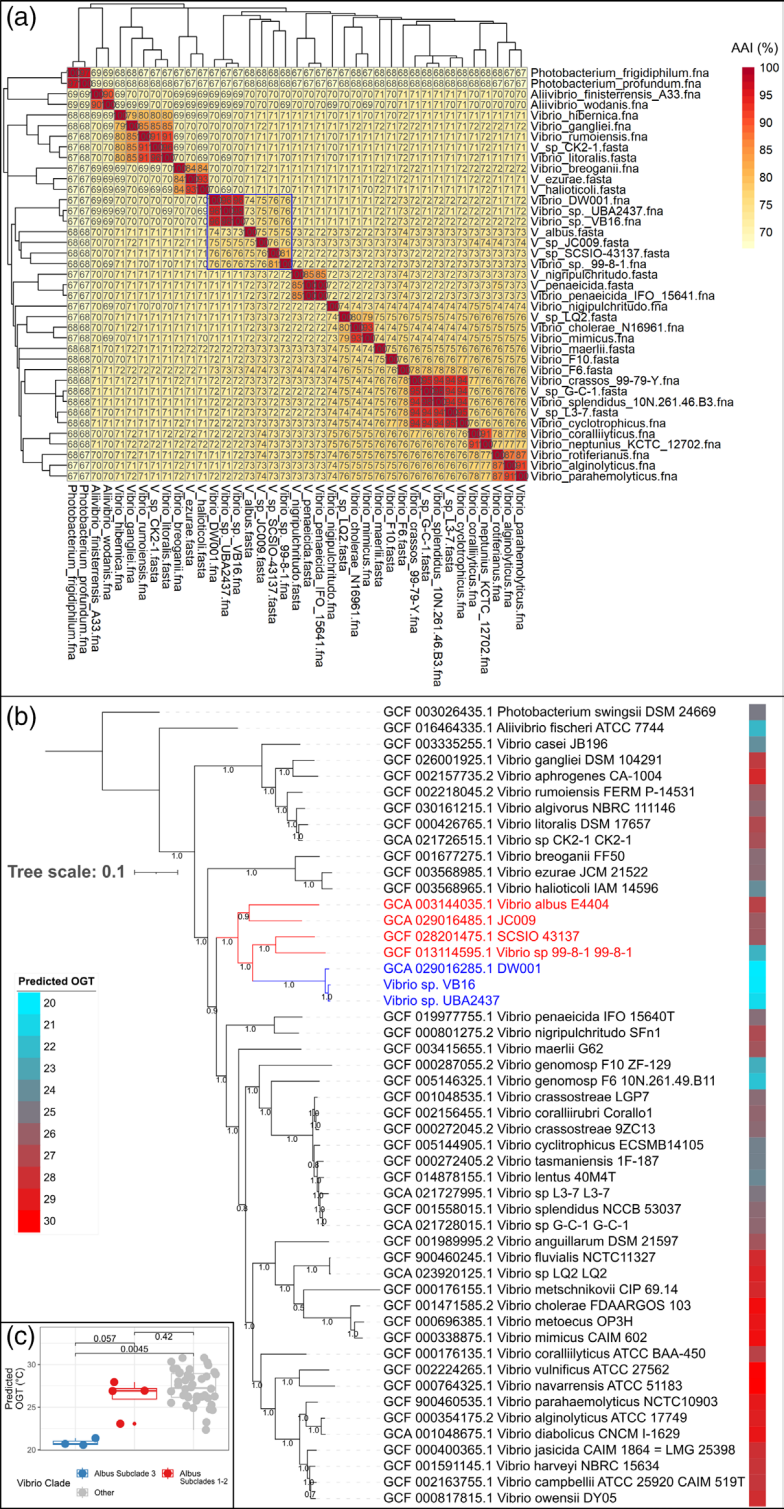
(**a**) An all vs all amino acid identity (AAI) heat map showing similarity in the amino acid sequence of the specified *Vibrio* genomes, including VB16. The blue box indicates the predicted *albus* clade. (**b**) A rooted, approximately maximum-likelihood phylogenomic tree constructed with VB16, related species and named *Vibrio* genomes from the NCBI database using single-copy marker genes from the Gammaproteobacteria set (172 genes). Numbers on each branch split refer to local support values calculated by FastTree. The tree was rooted using *Photobacterium swingsii* as the outgroup. Bar, 0.1 substitutions per nucleotide position. Proposed members of *albus* subclades 1 and 2 are shown in red on the tree, subclade 3 in blue. Heat map colours to the right of each node indicate the TOME predicted optimal growth temperatures for that genome in degrees Celsius. (**c**) Box plot comparing the TOME predicted optimal growth temperatures (OGTs) from the novel subclades of the *albus* clade to the other vibrios listed in (**b**). Numbers indicate the *P*-values from two-sided *t*-tests comparing the means of the groups.

We investigated whether *Vibrio* strains VB16, UBA2437 and DW001 clustered into a new clade. This was partially confirmed with an all vs all AAI matrix (Fig. 2a) using 37 *Vibrio* and 2 *Photobacterium* species, representing 10 recently described *Vibrionaceae* clades [20] (see Table S2 for a list of genomes used). *Vibrio* strains VB16, UBA2437 and DW001 formed a distinct cluster within this array of clades (AAI >95 %). However, three additional strains (99-8-1, SCSIO 43137, JC009) and one species (*V. albus*) formed a secondary cluster (blue box) with AAI values ranging from 73 to 81 % compared to VB16. *V. albus* and 99-8-1 have previously been shown to constitute single-member clades based on similar phylogenetic analyses using MLSA of large sets of single-copy marker genes [41, 42], but no other members of this potential clade have previously been phylogenetically placed.

Using the same vibrios from Fig. 2a, rooted and unrooted phylogenomic trees were constructed using GoTree based on multiple-sequence alignment of Gammaproteobacteria marker genes (172 genes). The seven genomes clustering from Fig. 2a grouped into the *albus* clade both in rooted (Fig. 2b) and unrooted trees (Fig. S1A). Based on our analysis, the newly expanded *albus* clade contains three branches near the root, consistent with three subclades (1, 2 and 3). A similar subclade structure was also observed using a tree generated with autoMLST (Fig. S1B). Our proposed, newly expanded *albus* clade was located between the *halioticoli* and *nigripulchritudo* clades. The placement of the *albus* clade next to *halioticoli* has been observed previously in unrooted *Vibrionacceae* trees found in Jiang *et al*. [20]. However, for reasons that likely relate to the different gene set used to construct the trees, the *nigripulchritudo* clade was placed closer to the *albus* clade in our trees compared to the trees found in Jiang *et al*. [20].

Using TOME, we predicted the OGT from the proteomes of *Vibrio* strains in subclades 1, 2 and 3 and compared to all other strains used to construct the phylogenomic tree in Fig. 1b. Most notably, the OGT of *albus* subclade 3 was lower than for subclades 2 and 3 (*P*=0.057, two-sided *t*-test) and significantly lower (*P*=0.0058, two-sided *t*-test) than for other vibrios, providing evidence of a novel cold-tolerant subclade (Fig. 2c). These bioinformatic findings were consistent with the different optimal growth temperatures measured experimentally for VB16 (subclade 3), which was 20 °C (Fig. 1) compared to 33 °C for *V. albus* (subclade 1) [41].

The proposed addition of new *Vibrio* strains into the *albus* clade (for accession numbers refer to Table S3) updates the most recent classification of *Vibrio* clades [20]. It is possible that other, as yet unsequenced, vibrios may also be members.

### Gene orthology in the *albus* clade and subclades

**Fig. 3.**
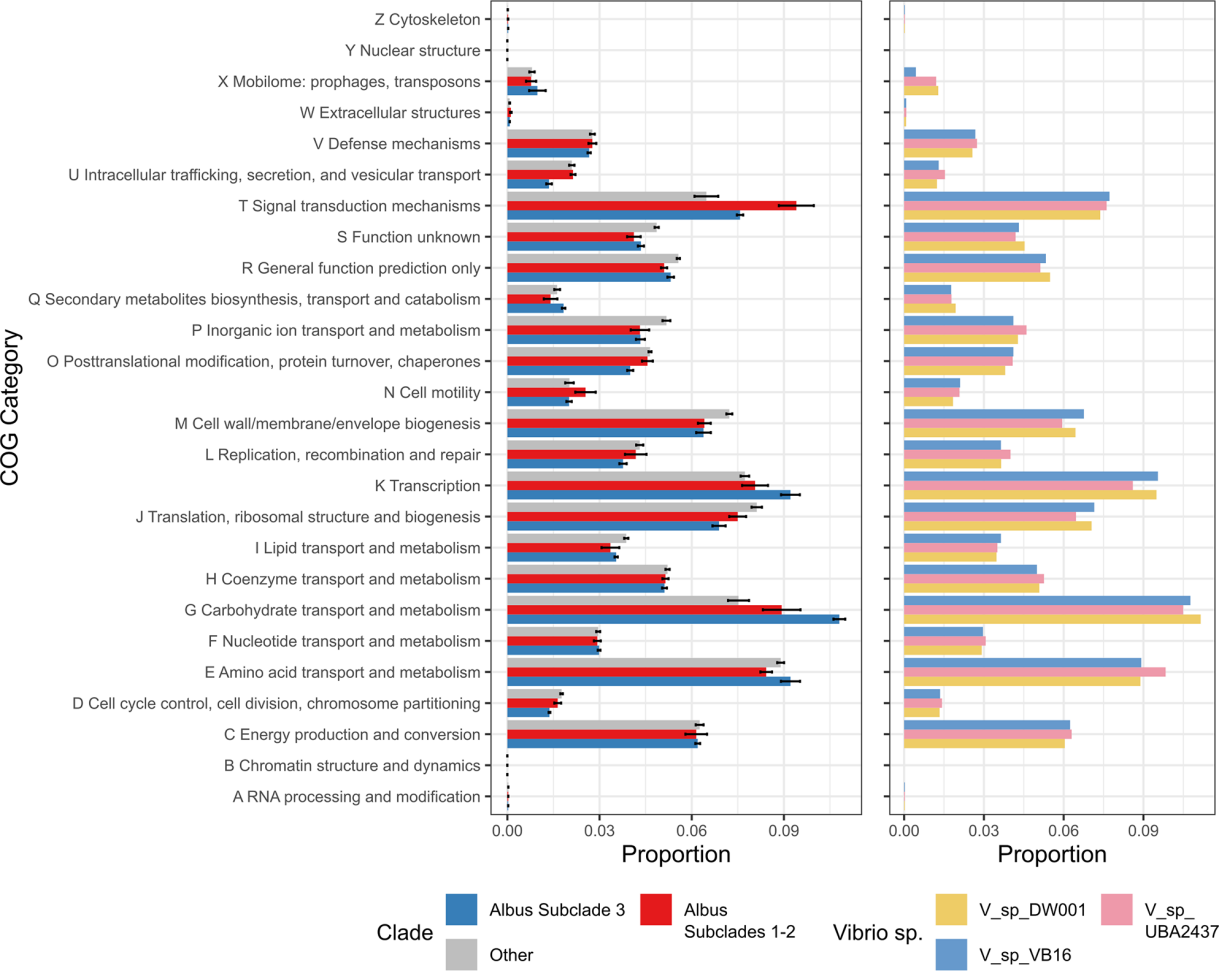
COG orthology of genomes from the *albus* subclades vs other *Vibrio* species from Fig. 2. Values are average proportions of genes in each category of all genomes; error bars are the standard error. On the right, comparison of COG orthologies of *Vibrio* genomes from *albus* subclade 3.

Next, we explored whether our proposed cold-tolerant subclade 3 had any distinguishing predicted functional features. Using COG categorization of genes in *Vibrio* genomes (as in Fig. 2), we found that subclade 3, compared to subclades 1 and 2, had greater numbers of genes for COG categories T (signal transduction mechanisms) and G (carbohydrate transport and metabolism) and fewer genes in categories P (inorganic ion transport and metabolism) and M (cell wall/membrane/envelope biogenesis) (Fig. 3, left). When comparing subclade 3 to all other vibrios, we found an enrichment for genes in categories K (transcription) and G (carbohydrate transport and metabolism). Within subclade 3, the number of genes in the various categories were similar across the three species/strains (Fig. 3, right).

**Fig. 4.**
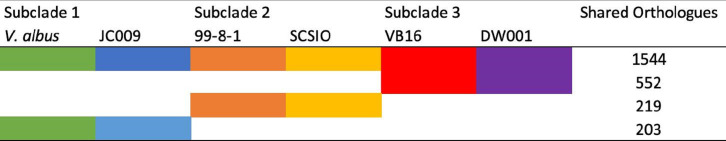
Protein orthologue clusters shared across the *albus* clade and three subclades. The UBA2437 genome was not included in this analysis given that it was only 90 % complete.

Orthovenn analysis indicated that VB16 and its 5 other *albus* clade members shared 1544 gene orthologue clusters (Fig. 4), ~45 % of the total orthologue clusters (average=3393) shared between these member genomes. Interrogation of these shared orthologues provided a glimpse into the potential lifestyle of *albus* clade members. We observed complete or near-complete pathways for the transport and metabolism of multiple carbohydrates, including some monosaccharides derived from polysaccharides produced specifically by eukaryotic organisms: fucose (a byproduct of fucoidan, found in mammalian intestinal tracts and plants, including seaweed [43]) and maltose (maltodextrin derived). We also observed genes for dissimilatory nitrate reduction (DNR: nitrate→nitrite). DNR is an anaerobic process [44], implying that members of this clade spend part of their life cycle in a high-organic-carbon environment with low oxygen levels, likely in marine sediments, consistent with the isolation conditions of VB16, UBA2437 and *V. albus*. We also found shared gene orthologues for nitrate/nitrite sensing, nitrite reduction to ammonia, reduction of nitrous oxide to nitrogen and nitrosative stress. Other shared orthologues included lifestyle genes involved with motility, adhesion, biofilm formation, defence and cold tolerance. This included a large multi-operon loci for flagellar assembly and movement, pili (TAD; type IV, MSHA; type II/IV) and cold shock proteins.

Of particular note is the presence of ethanolamine utilization genes within some *albus* clade members. Ethanolamine is an important source of carbon and/or nitrogen for bacteria prevalent in the gastrointestinal tract [45]. Subclade 1 shared orthologues for a four-gene ethanolamine utilization (eutBCHR) operon coding for the permease, lyase and regulatory protein enabling the conversion of ethanolamine into ammonia and acetaldehyde similar to that found *Vibrio alginolyticus* [46, 47]. Subclades 2 and 3 on the other hand, shared orthologues for an 18-gene *eut* operon coding for uptake and catabolism of ethanolamine to produce ethanol, ammonia, acetate and acetyl CoA, including the genes that form a bacterial microcompartment (BMC).

### Genes within subclade 3 promote cold tolerance, biofilm formation and adhesion

There were 552 orthologues unique to subclade 3 (Fig. 4); noteworthy among these are genes involved in the cold-tolerant phenotype or in a predicted lifestyle of colonizing a host gastrointestinal tract.

Among these shared orthologues was the five-gene eicosapentaenoic acid biosynthetic gene cluster. Increased synthesis of this polyunsaturated fatty acid is known to increase the membrane fluidity of bacteria, promoting low temperature tolerance. This gene cluster has only been found in the *splendidus* clade among all other vibrios [48]. Additionally, we found a single orthologue of the *litR* transcriptional repressor gene. In *Aliivibrio salmonicida*, LitR expression at low temperatures increases biofilm formation through the symbiosis polysaccharide (*syp*) locus [49], an important trait of psychrotolerant species [48]. The *syp* locus was unique to VB16, DW001 (though also found in 99-8-1). Other potential cold-tolerant adaptations included shared orthologues for novel giant proteins (PEG 1622 and PEG 4710 in VB16). These genes code for a secreted adhesin-like protein and contain one or more ice-binding domains (IBDs) [50]. Since the DW001 strain was isolated directly from Antarctic sea ice, this points to a potential role for an ice-binding protein to promote habitat selection for members of this subclade during freezing temperatures.

Subclade 3 genomes also contained shared orthologues for type I pilus and curli fibre synthesis, as well as a novel giant protein (~8000 amino acids) containing an N terminal LEPR-XLL domain and C-terminal RTX toxin and seralysin domains. Similar secreted proteins have been shown to function as a pore-forming cytotoxins, which could be important adaptations to colonizing a host gastrointestinal tract [51, 52].

## Conclusion

In this study, we have presented the genome of a novel cold-tolerant *Vibrio* strain, isolated from intertidal sediment during winter. We argue that this strain constitutes a new subclade within the *Vibrio albus* clade, consisting of three strains: strains VB16 and UBA2437 (predicted to be same species), and DW001 (Antarctic sea ice). This subclade represents a novel group of cold-adapted vibrios, as evidenced by growth experiments, predicted optimal growth temperatures and multiple genomic hallmarks of psychrotolerant or ‘moderate pyschrophile’ species. These species also appear to be adapted to multiple lifestyles, either free-living in marine sediments or as an opportunistic pathogen within a marine eukaryotic host. The discovery of this subclade opens the door for experimental work on the physiological features underpinning cold adaptation (lipid profiling, transcriptomic work, etc.) and the ecological importance of this subclade [potential eukaryotic host(s), distribution and abundance patterns over four seasons].

## Supplementary Data

Supplementary material 1
