# Supplementary material for: Complete genome sequence of a winter season Vibrio facilitates discovery of a novel subclade of cold-adapted species in the albus clade
Source: Microb Genom. 2024 Jan 17;10(1):001178. doi: 10.1099/mgen.0.001178 (PMC10868602; doi:10.1099/mgen.0.001178)
Supplement: Supplementary material 1 [file mgen-10-1178-s001.pdf]

Supplemental Tables and Figures

Table S1. List of genomes used for the GoTree phylogenomic tree and AAI matrix analyses.
* New in this study.

Genbank or Refseq Accession	Number of SCG hits used in final alignment	Species
GCA_001048675.1	158	*Vibrio diabolicus*
GCA_029016285.1	166	*Vibrio* sp. DW001
GCF_000176135.1	168	*Vibrio coralliilyticus*
GCF_000176155.1	170	*Vibrio metschnikovii*
GCF_000272045.2	160	*Vibrio crassostreae*
GCF_000272405.2	159	*Vibrio tasmaniensis*
GCF_000287055.2	167	*Vibrio genomosp. F10*
GCF_000338875.1	166	*Vibrio mimicus*
GCF_000354175.2	165	*Vibrio alginolyticus*
GCF_000400365.1	160	*Vibrio jasicida*
GCF_000426765.1	168	*Vibrio litoralis*
GCF_000696385.1	148	*Vibrio metoecus*
GCF_000764325.1	170	*Vibrio navarrensis*
GCF_000817815.1	159	*Vibrio owensii*
GCF_001048535.1	162	*Vibrio crassostreae*
GCF_001471585.2	170	*Vibrio cholerae*
GCF_001558015.1	160	*Vibrio splendidus*
GCF_001591145.1	161	*Vibrio harveyi*
GCF_001677275.1	157	*Vibrio breoganii*
GCF_001989995.2	167	*Vibrio anguillarum*
GCF_002156455.1	161	*Vibrio coralliirubri*
GCF_002157735.2	167	*Vibrio aphrogenes*
GCF_002163755.1	159	*Vibrio campbellii*
GCF_002218045.2	166	*Vibrio rumoiensis*
GCF_002224265.1	167	*Vibrio vulnificus*
GCF_003026435.1	158	*Photobacterium swingsii*
GCF_003335255.1	168	*Vibrio casei*
GCF_003415655.1	167	*Vibrio maerlii*
GCF_005144905.1	160	*Vibrio cyclitrophicus*
GCF_005146325.1	170	*Vibrio genomosp. F6*
GCF_013114595.1	160	*Vibrio* sp. 99-8-1
GCF_014878155.1	158	*Vibrio lentus*
GCF_016464335.1	162	*Aliivibrio fischeri*
GCF_026001925.1	168	*Vibrio gangliei*
GCF_030161215.1	168	*Vibrio algivorus*
GCF_900460245.1	159	*Vibrio fluvialis*
GCF_900460535.1	168	*Vibrio parahaemolyticus*
GCA_002342735.1	135	*Vibrio* sp. UBA2437
GCF_015594925.2	163	*Vibrio* sp. VB16*

Table S2. Genome quality assessment from QUAST and BUSCO analysis of the VB16 genome

Statistics	VB16 assembly
Largest contig	2
# contigs (>=0 bp)	2
# contigs (>=1,000 bp)	2
# contigs (>=5,000 bp)	2
# contigs (>=10,000 bp)	2
# contigs (>=25,000 bp)	2
# contigs (>=50,000 bp)	2
Total length (>=0 bp)	5,210,103
Total length (>=1,000 bp)	5,210,103
Total length (>=5,000 bp)	5,210,103
Total length (>=10,000 bp)	5,210,103
Total length (>=25,000 bp)	5,210,103
Total length (>=50,000 bp)	5,210,103
Total # contigs	2
Largest contig	3,636,985
Total Length	5,210,103
GC (%)	41.87
N50	3,636,985
N75	1,573,118
L50	1
# N's/100 kbp	0
BUSCO groups searched	
Complete BUSCOs (C)	1422 (98.5%)
Complete and Single Copy BUSCOs (S)	1414 (97.9%)
Complete and Duplicated BUSCOs (D)	8 (0.6%
Fragmented BUSCOs	2 (0.1%)
Missing BUSCOS	21 (1.4%)
Total BUSCO groups searched	1445 (100%)

Table S3. Accession numbers for Albus clade genomes. MAG; metagenomic assembled genome

Strain/species Genome	Genome Assembly Status	NCBI accession number	Number contigs	Contig N50 (kbp)	Citation
V. albus	contig	GCA_003144035.1	37	353.5	41
JC009	complete	GCA_029016485.1			
99-8-1	contig	GCF_013114595.1	85	126.1	42
SCSIO 43137	complete	GCF_028201475.1			
UBA2437	contig (MAG)	GCA_002342735.1	690	10.4	39, 40
DW001	complete	GCA_029016285.1			
VB16	complete	GCF_015594925.2			this study

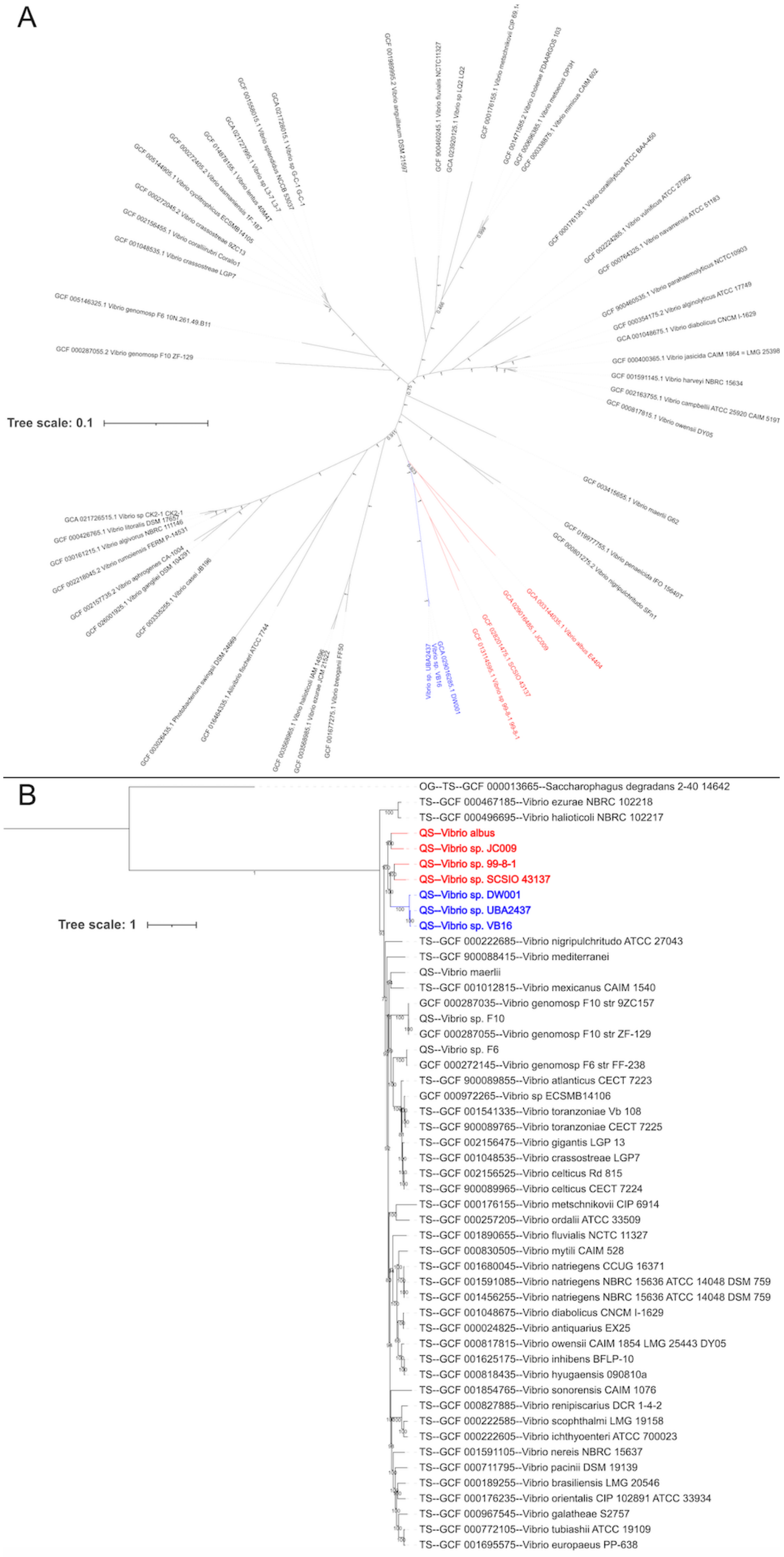

Figure S1. (A) Unrooted phylogenetic tree, constructed similarly to Figure 2B but without a root (using GoTree; refer to Methods). Members of proposed Albus subclades 1 and 2 are shown in red, subclade 3 is shown in blue. (B) A rooted phylogenetic tree, constructed using autoMLST, which automatically adds species to the tree based on similarity to a set of query sequences (refer to Methods). The tree is based on multiple-locus sequence alignment of 78 genes.
